# Addressing comprehensive complexities a striking familial hypercholesterolemia case study

**DOI:** 10.1186/s43044-024-00483-5

**Published:** 2024-04-20

**Authors:** Shazia Rasheed, Ghulam Kubra, Lubna Baqai, Muhammad Liaquat Raza, Fariha Hassan, Syed Ghazi Abbas Rizvi

**Affiliations:** 1 Non-Invasive Cardiology at National Institute of Cardiovascular Diseases (NICVD), Rafiqui (H.J.) Shaheed Road, Karachi, 75510 Pakistan; 2 Tabba Heart Institute, Karachi, Pakistan; 3 Al Fatima Hospital, Karachi, Pakistan; 4 Department of Infection Prevention Control, Ministry of National Guard Health Affairs, Riyadh, Saudi Arabia; 5 King Abdullah International Medical Research Center, Riyadh, Saudi Arabia; 6 King Saud Bin Abdulaziz University for Health Sciences, Riyadh, Saudi Arabia

**Keywords:** Familial hypercholesterolemia, Coronary artery disease, Aortic stenosis, Case report

## Abstract

**Background:**

Premature aortic involvement and comprehensive management strategies in familial hypercholesterolemia familial hypercholesterolemia (FH), a rare autosomal dominant genetic disorder, poses significant challenges due to its propensity for elevated low-density lipoprotein cholesterol, premature coronary heart disease, and vascular atherosclerosis.

**Case presentation:**

Unraveling Cardiovascular Complexities: A Striking Familial Hypercholesterolemia. This case study delves into a remarkable instance of FH in a 16-year-old female who presented with chest pain and worsening dyspnea. Diagnostic evaluation revealed distinct electrocardiographic changes, elevated troponin levels, and profound dyslipidemia. Remarkable findings on transthoracic echocardiography, computed tomography angiography, and catheterization prompted multidisciplinary interventions and demonstrated remarkable enhancements in ventricular function, mitral regurgitation, and aortic stenosis.

**Conclusion:**

The case study underscores the urgency of comprehensive management strategies in confronting the myriad challenges of FH, emphasizing the value of early intervention, innovative therapies, and rigorous imaging modalities for unraveling the intricate cardiovascular manifestations of this condition.

## Background

Familial hypercholesterolemia (FH) is an autosomal dominant genetic disorder characterized by elevated low-density lipoprotein cholesterol (LDL-C), xanthoma formation, premature coronary heart disease, and vascular atherosclerosis, primarily attributed to mutations in the LDL receptor gene [1]. FH is a rare condition, affecting only one in a million individuals [2, 3]. This disorder contributes to accelerated atherogenesis, particularly impacting the aortic system and potentially leading to aortic root narrowing [3].

Furthermore, early-onset coronary atherosclerosis resulting in ischemic heart disease imposes a substantial burden, prompting acute coronary events at a younger age. Notably, FH-associated complications encompass aortic root involvement, supravalvular aortic stenosis, premature coronary ostial stenosis, and a significantly elevated risk of morbidity and mortality in the youthful population [4–6].

## Case presentation

**Fig. 1:**
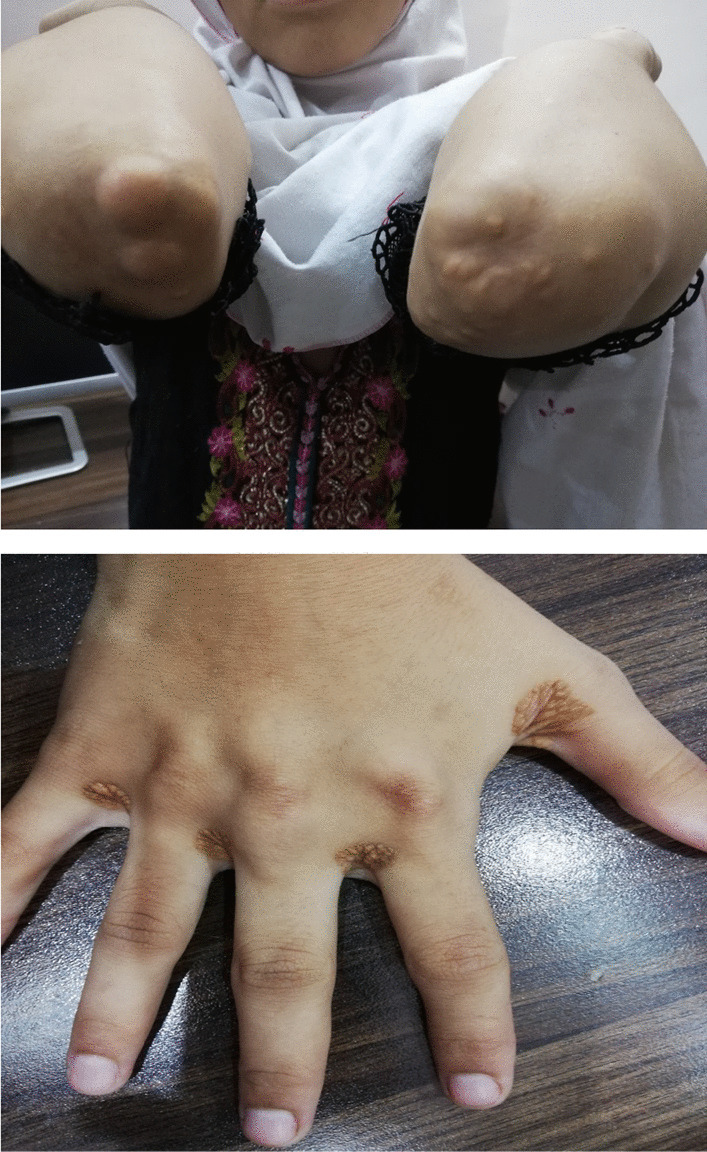
Cutaneous xanthelasmas on extensor surface

**Fig. 2:**
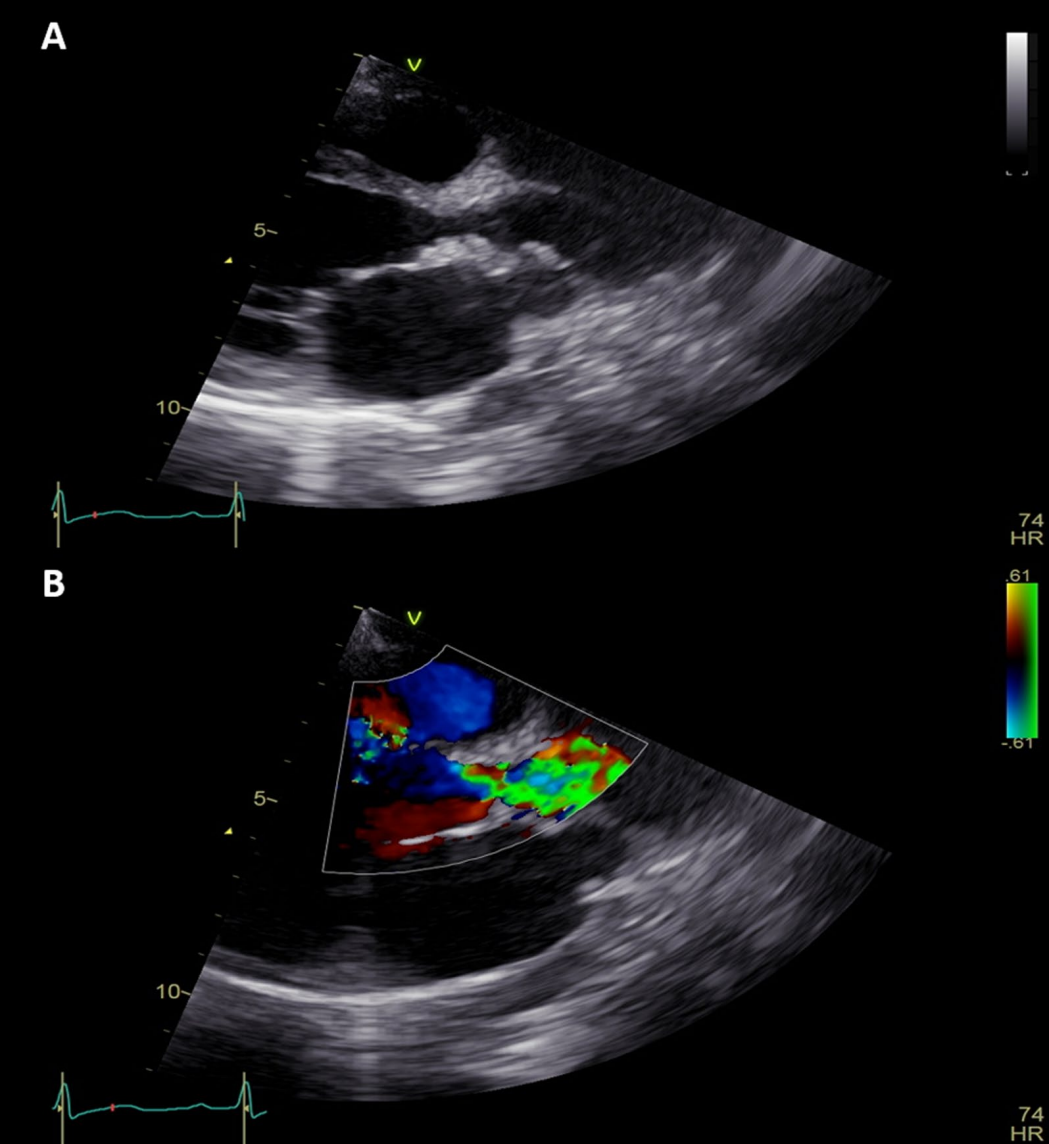
**a** Parasternal long axis view showing narrowed aortic root on 2D, and **b** turbulence on color Doppler

**Fig. 3:**
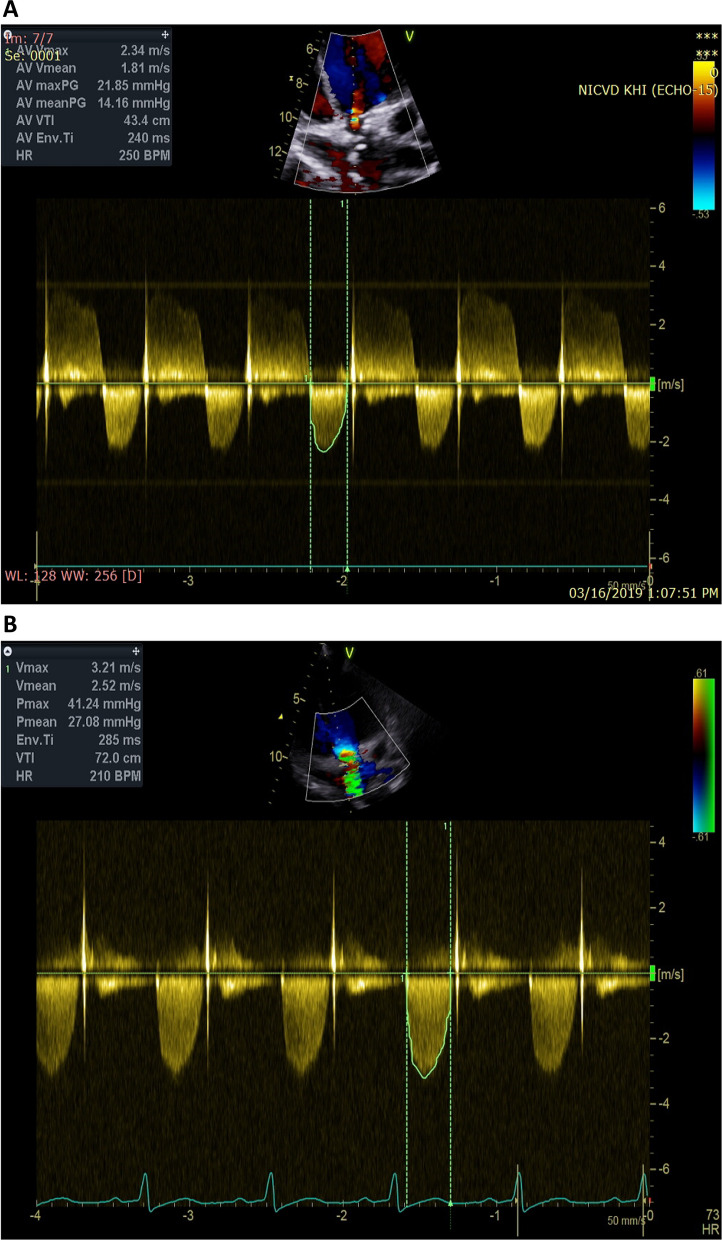
**a** Continuous wave Doppler on TTE showing gradient across Aortic valve, **b** which increases after coronary revascularization after increasing in LV systolic function

**Fig. 4:**
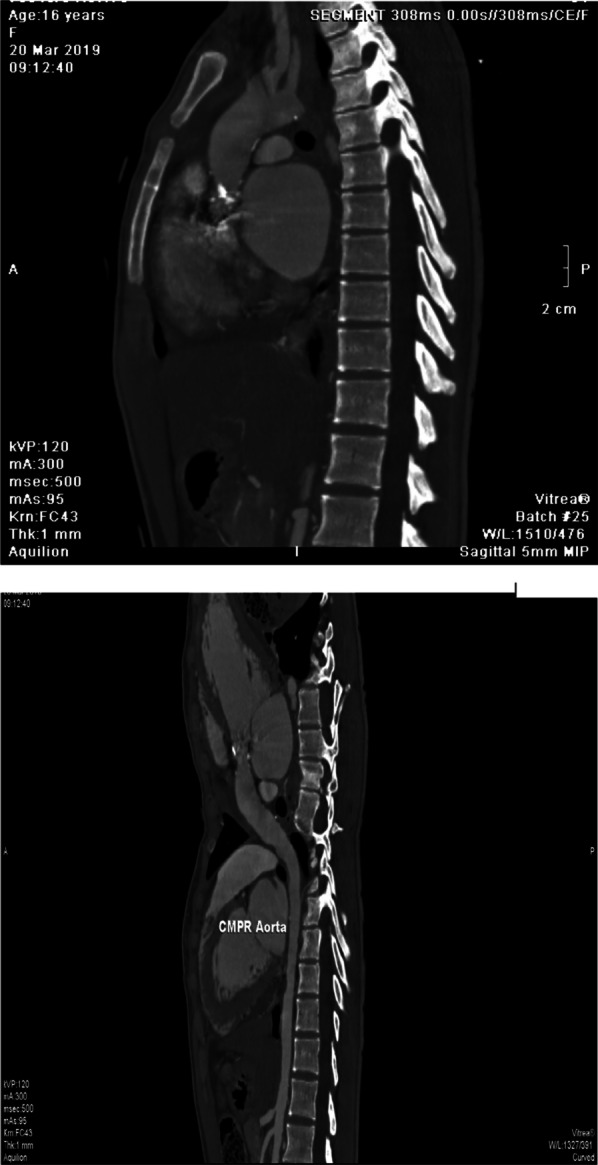
CT aortogram showing significant narrowing of aorta

**Fig. 5:**
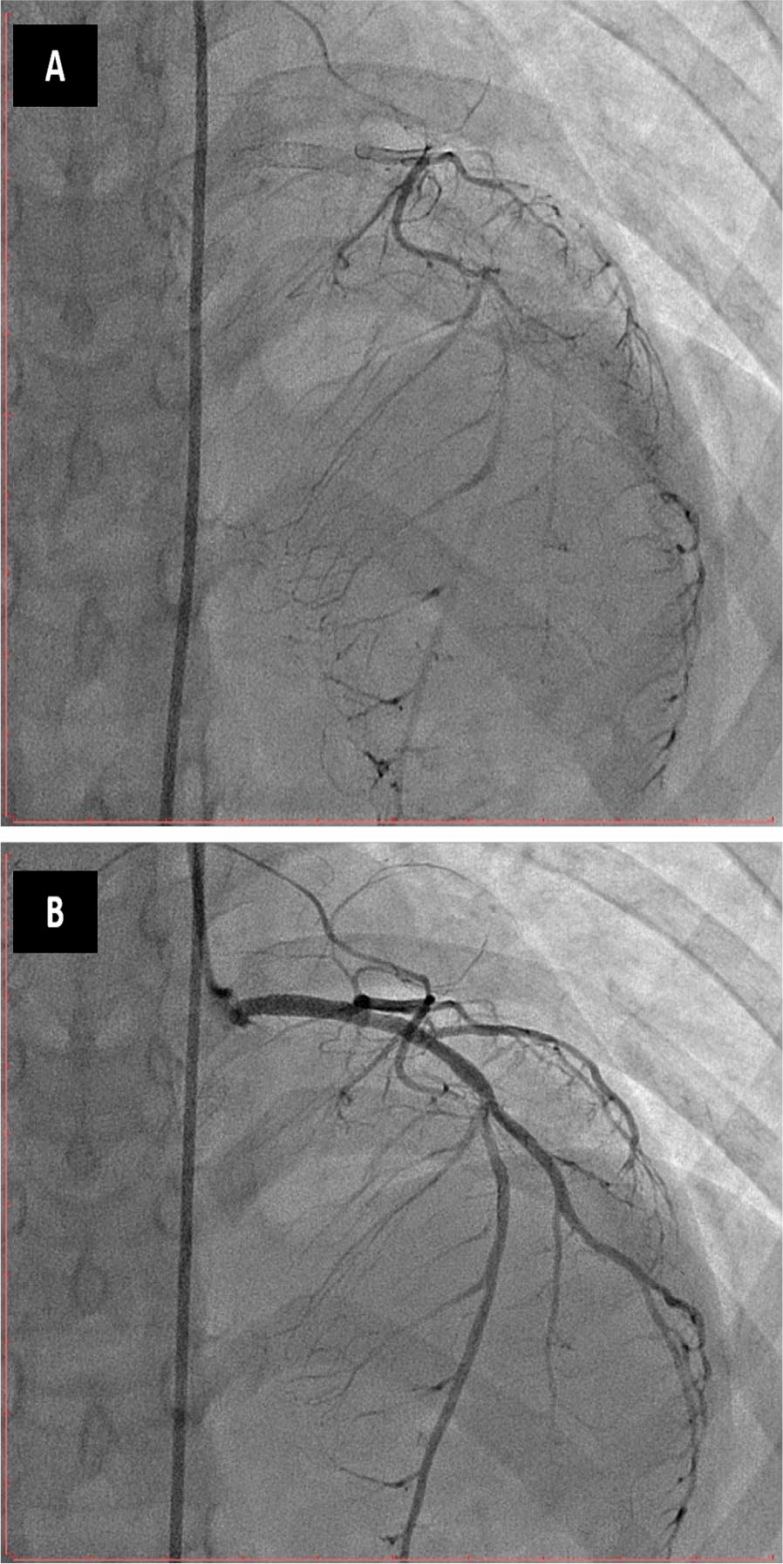
Successful revascularization of ostial left anterior descending artery (LAD) and ostial right coronary artery (RCA)

In this case study, we present a 16-year-old female with a confirmed diagnosis of familial hypercholesterolemia who sought emergency care due to chest pain and deteriorating dyspnea. Upon cardiac auscultation, a distinct ejection systolic murmur was detected along the right upper sternal border. Physical examination revealed cutaneous xanthomas on the hands, elbows, and legs (Fig. 1, informed written consent secured for publication). Electrocardiogram (ECG) analysis displayed inadequate progression of R waves in anterior leads, coupled with ST segment depression and T wave inversion in lateral leads. Elevated troponin I levels (17.15 ng/dL) were observed, alongside dyslipidemia characterized by total cholesterol of 569 mg/dL, HDL of 59 mg/dL, LDL-C of 484 mg/dL, and triglycerides at 129 mg/dL. Remaining laboratory parameters were unremarkable. Transthoracic echocardiography revealed severe generalized left ventricular systolic dysfunction (EF: 20%), profound secondary mitral regurgitation, and marked aortic root narrowing (Figs. 2, 3a) with mean and peak pressure gradients of 14 and 22 mmHg, respectively. Computed tomography angiography verified these findings, showcasing a thickened aortic wall with calcification in the ascending aorta. Distinct wall thickening encompassed coronary ostia and branches of the aortic arch. Aortic measurements were: Annulus 14.9 mm, Sinus 10.6 mm, STJ 11.5 mm, ascending aorta 19.8 mm, Arch 18.3 mm, descending thoracic aorta 13.2 mm, and abdominal aorta 7.8 mm (Fig. 4). Left heart catheterization demonstrated significant ostial stenosis in the left anterior descending (LAD) and right coronary arteries. After multidisciplinary consultation, the patient underwent percutaneous coronary intervention for LAD and right coronary artery (Fig. 5). Subsequent echocardiography revealed enhanced left ventricular systolic function (EF: 40%), reduced mitral regurgitation, and notably, an increased supravalvular stenosis gradient (Fig. 3b). The patient exhibited marked symptom improvement following revascularization, combined with optimized medical therapy involving high-dose statin and ezetimibe. Follow-up after 1 month indicated asymptomatic status and regular clinical monitoring.

## Discussion

The management of familial hypercholesterolemia encompasses diverse strategies. Robust evidence supports the effectiveness of high-dose statin therapy alongside ezetimibe for substantial LDL reduction [7]. For refractory cases, lipid apheresis and innovative proprotein convertase subtilisin/kexin type 9 (PCSK9) inhibitors, such as alirocumab and evolocumab, offer promising alternatives. PCSK9 inhibitors augment LDL receptor availability, promoting enhanced LDL absorption and consequent reduction in cardiovascular risk [7]. Noninvasive imaging plays a pivotal role in evaluating cardiac and aortic involvement. Techniques encompass echocardiography, magnetic resonance imaging (MRI), and CT scans, each offering detailed insights into the heart, aorta, and coronary arteries [8]. Echocardiography gauges valvular function and blood flow, while CT scans detect coronary calcification indicative of atherosclerosis. Left heart catheterization guides treatment planning by delineating coronary involvement. Management options, tailored to disease severity and patient characteristics, span percutaneous stenting, conventional coronary artery bypass grafting, surgical interventions, and hybrid approaches [9, 10].

## Conclusion

This comprehensive analysis underscores the significance of addressing cholesterol disorders in early stages to mitigate substantial morbidity and mortality. Rigorous assessment, employing noninvasive and invasive modalities, is imperative for unraveling FH-related complications. Novel therapeutic agents warrant consideration to curtail disease progression.

## Data Availability

Not applicable.

## Abbreviations

CT: Computed tomography
ECG: Electrocardiogram
FH: Familial hypercholesterolemia
LAD: Left anterior descending
LDL-C: Low-density lipoprotein cholesterol
LV: Left ventricle
MRI: Magnetic resonance imaging
PCSK9: Proprotein convertase subtilisin/kexin type 9
